# Lung Ultrasonography Does Not Distinguish between Interstitial and Alveolar Pulmonary Edema

**DOI:** 10.3390/diagnostics14030324

**Published:** 2024-02-02

**Authors:** Jing Liu

**Affiliations:** Department of Neonatology and NICU, Beijing Obstetrics and Gynecology Hospital, Capital Medical University, Beijing 100026, China; liujingbj@live.cn

## 1. Introduction

For a long time, lung diseases have been considered the “forbidden zone” for ultrasound diagnosis because the lung is filled with gas, and the ultrasound waves are totally reflected when they encounter gas. However, in the past 20 years, this “forbidden zone” has been broken, and lung ultrasonography (LUS) has been used for the diagnosis and differential diagnosis of various lung diseases [1,2,3,4,5,6]. Especially in the diagnosis and differential diagnosis of neonatal lung diseases, LUS has higher accuracy and reliability than chest X-ray (CXR) [7,8,9,10]. Animal experimental studies have also found that its accuracy and reliability are comparable to the results of chest CT examination in the diagnosis of a variety of lung diseases, such as pneumothorax, pulmonary edema, lung consolidation, or atelectasis [11,12]. In addition, there is no associated radiation damage, and it can be performed at the bedside, which is convenient for dynamic observation and does not affect the rescue and treatment of critically ill patients. Therefore, an increasing number of clinical applications have been made, neonatal radiation exposure has been significantly reduced, and even CXR examination has been completely replaced to open a new “era of green diagnosis” of neonatal lung diseases, steering away from methods that cause radiation damage [13,14]. However, whether LUS can accurately distinguish between alveolar and interstitial pulmonary edema remains controversial.

## 2. The Definition and Classification of B-Lines in LUS

Combined with the literature, an international expert consensus group has given the following definition and classification of the B-line [3,15]: the B-line is an artifact caused by the reflection of ultrasound waves encountering the air–liquid interface in the lungs. On the ultrasound, it is manifested as a series of linear hyperechoic reflections, originating from and perpendicular to the pleural line, spreading radially to the deep part of the lung fields and reaching the edge of the scanning screen without attenuation. When the probe is scanned perpendicular to the ribs, if the B-lines are densely present in the whole intercostal space, that is, the B-lines are fused with each other and it is difficult to distinguish them from one another and to count, such B-lines are called confluent B-lines. Alveolar-interstitial syndrome (AIS) is defined as the presence of confluent B-lines in more than two consecutive intercostal spaces in any scanning area, but the acoustic shadow of the ribs is still clearly displayed. When the probe is scanned vertically with the ribs, the acoustic shadow of the ribs in the whole scanning area disappears due to the existence of a dense B-line, which is called a compact B-line. When all lung fields present as compact B-lines, this is known as white lung. It is generally believed that pulmonary edema is indicated when there are many B-lines in the lung fields, while the confluent B-line, compact B-line, and white lung are all manifestations of severe pulmonary edema.

## 3. LUS Can Achieve Accurate Diagnosis and Quantitative Assessment of Pulmonary Edema Based on B-Lines

Pulmonary edema usually refers to the pathological accumulation of extravascular lung water (EVLW), in which fluid exudation exceeds the physiological fluid flow from blood vessels to the stroma due to an imbalance between exudation and absorption. Under physiological conditions, the filtrate is reabsorbed mainly through lymphatic vessels located around the blood vessels of the bronchus, the interlobular septum, and the subpleural space. Pulmonary edema is caused when the clearance capacity of the lymphatic system is not insufficient to remove more filtrate from the interstitial space or/and impaired alveolar–capillary barrier or/and epithelial malabsorption happened, resulting in dysregulated fluid transport between the alveolar endothelium and epithelial cells.

LUS has definite diagnostic value for pulmonary edema [16,17,18,19,20], and LUS manifestations are closely related to clinical manifestations [19,20]. When LUS manifests as scattered B-lines or alveolar-interstitial syndrome (AIS), most infants present with mild dyspnea and an increased respiratory rate, generally as type I respiratory failure. When LUS manifests as confluent B-lines, patients often present with type II respiratory failure clinically and need to be treated with a noninvasive ventilator. However, when LUS manifests as compact B-lines or “white lung”, the patient not only has type II respiratory failure but also cardiac function may be seriously affected, and invasive ventilator treatment is needed at this time.

We established a model of alveolar pulmonary edema by injecting 0.9% NaCl into the lungs of experimental animals (rabbits) through tracheal intubation [19,20]. Forty-five New Zealand rabbits were divided into nine groups, and different amounts of 0.9% NaCl were injected into the lungs through tracheal intubation. Changes in LUS, respiration, heart rate, arterial blood gas, lung dry/wet ratio, and pathological changes were also detected. It was found that LUS showed different B-lines with different volumes of 0.9% NaCl injected into the lung, and the corresponding clinical symptoms also appeared different. For example, when the volume of injected 0.9% NaCl was <6 mL/kg body weight, LUS only showed the comet-tail signs or scattered B-lines, and the experimental animals had no obvious respiratory symptoms. When the volume of 0.9% NaCl was more than 10 mL/kg body weight, the LUS showed confluent B-lines, and the experimental animals showed obvious dyspnea, which manifested as type II respiratory failure. However, when the volume of injected 0.9% NaCl was 20 mL/kg body weight, the LUS showed compact B-lines or “white lung”, and the experimental animals showed not only type II respiratory failure but also significantly decreased heart rate or even cardiac arrest [19,20]. These results were also very close to the results of clinical research.

## 4. It Is Difficult to Make a Clear Judgment on the Properties of Pulmonary Edema by LUS

Nearly ten years ago, experts proposed the concept of the B3 line and B7 line [21]. It was believed that the distance between two B-lines can distinguish interstitial and alveolar pulmonary edema; that is, when the distance between two B-lines is ≤3.0 mm, it is called the B3 line, indicating alveolar pulmonary edema. However, when the distance between two B-lines is 6.0–7.0 mm, it is called the B7 line, indicating interstitial pulmonary edema [21]. This opinion is still adopted today [22]. However, an increasing number of studies have found that the morphology of B-lines is affected by many factors, such as different types of ultrasound instruments, probe types and probe frequency, scanning depth, numbers and position of focus, and spatial composite imaging function turning on or off, which can affect the expression of B-lines and the evaluation results of B-lines [23,24,25,26]. Japanese scholar Kameda et al. [23,24] conducted a study in humans when using a linear array probe with a frequency of 6 MHz, turning off the space composite imaging function and the edge enhancement and speckle reduction function, where an individual B-line was generated when the scanning depth was 10 cm and the focus position was close to the pleural line. When the other parameters were unchanged, the depth of the focus was adjusted to 4.5 cm, and the distal scattered B-line was generated. However, when the focus position was adjusted to a depth of 7 cm, the dispersion at the distal end of the B-line was more obvious. Similarly, other parameters remained unchanged. When the open space is compatible with imaging, the resulting B-line is dispersed into multiple lines at the distal (deep) end. A study jointly completed by scholars from the United States and Canada also found that scanning depth, gain, and focus position have a significant impact on B-lines [25]. They investigated the effect of scan depth, gain, focus position, and transducer type on the quality of B-lines images. Different video clips were acquired by varying the scan depth (6, 12, 18, and 24 cm for curvilinear transducer and 4 and 8 cm for linear probes), gain (10%, 50%, and 90%, respectively), and focus position (at the pleural line or half of the scan depth). B-lines and image quality were scored by clipping. The results showed that the curve transducer improves the quality of the B-lines. When the focus is directed toward the pleural line, the greater the gain, the better the visualization of the B-lines. Image quality was best at a gain of 50%, but it decreased when the gain increased to 90% and the focus was located at the pleural line. When other parameters were unchanged, the best image quality was obtained when the scanning depth was 12–18 cm. The B-line was best displayed using a curvilinear transducer with a gain of at least 50% and a focus position at the pleural line. A gain of less than 90% and a scan depth between 12 and 18 cm can improve image quality [25]. Matthias et al. [26], an American scholar, found that different probe types and different preset conditions (such as small organ conditions, heart conditions, abdominal conditions, etc.) selected during lung ultrasound examination had significant effects on B-line morphology and quality. Polish scholar Łobaczewski et al. [27] examined pulmonary edema in different kinds of animals (dogs and cats) using different models of probes and found they also have a significant impact on the form of B-lines and image quality. Because there are many factors that influence the performance and quality of B-lines, it is almost impossible to judge the properties of pulmonary edema based on the distance between B-lines.

## 5. Clinical and Experimental Studies Have Found That Interstitial, Alveolar, or Mixed Pulmonary Edemas Have the Same or Similar Ultrasound Findings

**Figure 1:**
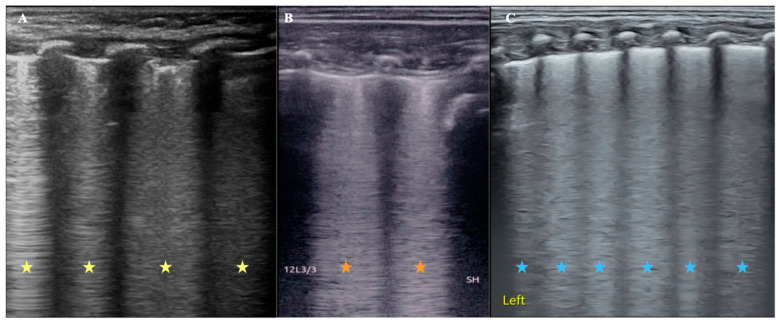
Comparison of ultrasound B-line manifestations of pulmonary edema with different properties. (**A**): An infant with viral pneumonia. LUS appears as confluent B-lines (yellow asterisk). (**B**): Rabbit model of pulmonary edema. Because LUS examination was performed immediately after 0.9% NaCl was injected into the lung from the endotracheal tube, the kind of pulmonary edema should be mainly alveolar pulmonary edema at this condition, and LUS appeared as confluent B-lines (brick-red asterisk). (**C**): Infant with wet lung. The mixed pulmonary edema and LUS also showed confluent B-lines. It is difficult to distinguish the type of pulmonary edema from the appearance of the B-lines (blue asterisk).

It is well known that viral pneumonia is mainly a kind of interstitial disease, and its pulmonary edema is mainly interstitial edema, while the wet lung of a newborn is mixed pulmonary edema. As mentioned above, we established a rabbit model of alveolar pulmonary edema by instillation of 0.9% NaCl through the endotracheal tube [19,20]. In these cases, severe pulmonary edema of varying nature is found on ultrasonography as confluent B-lines [Figure 1], which can develop into enigmatic B-lines or white lungs. Figure 1 shows that pulmonary edema, whether alveolar, interstitial, or mixed, has the same or similar appearance on ultrasound.

## 6. It Is also Difficult to Distinguish Cardiogenic Pulmonary Edema from Pulmonary Lung Edema Using Lung Ultrasound

Cardiogenic pulmonary edema can be a life-threatening condition and a common cause of respiratory failure in emergency medicine; both animal experiments and clinical studies have confirmed that LUS can cause cardiogenic pulmonary edema [16,28,29]. Based on pathophysiological mechanisms, cardiogenic pulmonary edema is mainly manifested as interstitial pulmonary edema in the early stage and can further cause alveolar pulmonary edema and mixed pulmonary edema as the disease deteriorates. As mentioned above, pulmonary edema of any property appears as a B-line increasing on the ultrasound image (Figure 1), just the type of B-line varies with the degree of pulmonary edema. Therefore, strictly speaking, LUS cannot distinguish between cardiogenic and pulmonary lung edema. However, since the main cause of cardiogenic pulmonary edema is left heart failure, we can distinguish this using cardiac ultrasound.

## 7. Conclusions

In conclusion, numerous clinical and experimental studies have confirmed that the shape and space between B-lines are affected by many factors [30]. The distance between B-lines reflects the degree of B-line fusion, which is related to the lung water content and the degree of pulmonary edema but not to interstitial or alveolar edema. Therefore, there is insufficient evidence to distinguish interstitial pulmonary edema or alveolar pulmonary edema based on the distance between the two B-lines [17,31,32]. Our animal experimental research results and long-term clinical practice have also shown that the accuracy of ultrasound examination results is also affected by many factors, not only B-lines, so we formulated the operation specifications and guidelines for LUS examination to ensure the accuracy, reliability, and comparability of examination results [33]. Next, more accurate animal experiments are needed to study the various possible influencing factors of B-lines and to further clarify the correlation between B-line performance and pulmonary edema, so as to better serve clinical practice.

## Abbreviations

LUS: lung ultrasonography; CXR: chest X-ray; EVLW: extravascular lung water; AIS: alveolar-interstitial syndrome; CPE: cardiogenic pulmonary edema.
